# Avian Influenza and US TV News

**DOI:** 10.3201/eid1211.060672

**Published:** 2006-11

**Authors:** Brian G. Southwell, Yoori Hwang, Alicia Torres

**Affiliations:** * University of Minnesota, Minneapolis, Minnesota, USA;; † American Institute of Physics, College Park, Maryland, USA

**Keywords:** Communication, influenza in birds, mass media, television news, letter

To the Editor: Scholars have routinely noted ways in which scientific inquiry is isolated from public life and popular attention and have bemoaned relatively low levels of scientific literacy among lay audiences (*1**–**3*). While public understanding of science in the United States and elsewhere undoubtedly is not at the level desired by most scientists, apparent interest and hunger to learn are high for certain issues. These issues represent public communication opportunities.

Avian influenza is now such an issue. Although the risk for pandemic human influenza stemming from the avian influenza H5N1 virus is thought to be relatively low (*4*), media coverage of the disease, at least superficial and episodic coverage of disease incidence, has been dramatic. Aside from existing coverage, however, what type of coverage should the issue receive according to viewers? Are they interested in the issue, if at all, as a matter of scientific inquiry or simply as a sensational threat to individual survival?

We report here relevant results from a national survey of local television news viewers in the United States. Evidence from an Internet-based survey conducted in May 2006 suggests that viewers not only think that the potential direct impact of avian influenza on their own lives should be covered by reporters but also have interest in scientific investigation of the disease.

Working with Survey Sampling International (available from http://www.surveysampling.com), we recruited by email a nationally representative sample of regular television news viewers. Potential respondents were offered the chance to win a cash prize. Only those >18 years of age and those who watched local television news show at least twice a week in recent months contributed to the final survey. We report here data from the 2,552 respondents who met those criteria and who answered all relevant questions.

Participants represented a reasonable cross-section of the general US population of television news viewers. Participants were 18–90 years of age (mean age 52, SD = 15.45). Educational attainment was mixed: 37% reported having completed at least a 4-year undergraduate degree, and 63% had completed <4-year degree. The final sample was 87% Caucasian, 8% African American, and 2% Asian; 8% also identified themselves as Hispanic or Latino. Approximately 54% of the sample was female, and 11% reported that they work for an organization directly involved in science.

When offered a 7-point scale that ranged from "not at all important" to "very important" to describe the priority that local television news should assign to addressing the "direct impact" of avian flu on one's own life and the lives of others, ≈80% chose >5. Approximately 42% of respondents chose the highest level, indicating it was very important for local television news to cover this angle of the story. Regarding deeper perspectives on the story, ≈81% of respondents chose >5 on the 7-point scale of importance when asked about potential coverage of how avian flu spreads and why scientists are finding it difficult to contain; 41% of respondents thought that it was "very important" that television reporters explicitly discuss that aspect of the issue. Moreover, 69% of respondents, by offering >5 on the 7-point scale, thought the television news should focus on the connection of avian flu to other issues, such as business and travel. Clearly, we are living in a time in which news audiences would tolerate much more than the soundbites and superficial coverage often offered with regard to infectious disease research.

Equally as striking are the demographic characteristics of those who believe that local television news should cover the process of scientific discovery in this arena. We conducted a simple regression analysis to predict 1 of the items noted above, i.e., perceived importance of television news discussion of how avian flu spreads and of the efforts of scientists. We used formal employment with a scientific institution, level of educational attainment (a 5-level variable treated here as interval), and reported conversation with others about science in recent months as predictors. Educational attainment actually bore a negative relationship to interest in such coverage, β = -0.14, p<0.01, and formal affiliation with a scientific institution bore no statistically significant relationship, p>0.10. (Past conversation about science bore a positive relationship, β = 0.06, p<0.01.)

Results suggested a prime opportunity for public communication efforts not just because of issue timeliness but also because of apparent widespread hunger for information among the US television news viewers. Health and science communication professionals could address this interest and desire to boost popular awareness of epidemiologic and medical inquiry.
